# Rapid crossed responses in an intrinsic hand muscle during perturbed bimanual movements

**DOI:** 10.1152/jn.00282.2019

**Published:** 2019-12-18

**Authors:** Katie Y. W. Khong, Ferran Galán, Demetris S. Soteropoulos

**Affiliations:** ^1^Institute of Neuroscience, Newcastle University, Newcastle upon Tyne, United Kingdom; ^2^Department of Basic Neurosciences, Faculty of Medicine, University of Geneva, Geneva, Switzerland; ^3^Queen's University Belfast, Belfast, Northern Ireland

**Keywords:** bimanual, crossed, hand, muscle, perturbation

## Abstract

Mechanical perturbations in one upper limb often elicit corrective responses in both the perturbed as well as its contralateral and unperturbed counterpart. These crossed corrective responses have been shown to be sensitive to the bimanual requirements of the perturbation, but crossed responses (CRs) in hand muscles are far less well studied. Here, we investigate corrective CRs in an intrinsic hand muscle, the first dorsal interosseous (1DI), to clockwise and anticlockwise mechanical perturbations to the contralateral index finger while participants performed a bimanual finger abduction task. We found that the CRs in the unperturbed 1DI were sensitive to the direction of the perturbation of the contralateral index finger. However, the size of the CRs was not sensitive to the amplitude of the contralateral perturbation nor its context within the bimanual task. The onset latency of the CRs was too fast to be purely transcortical (<70 ms) in 12/12 participants. This confirms that during isolated bimanual finger movements, sensory feedback from one hand can influence the other, but the pathways mediating the earliest components of this interaction are likely to involve subcortical systems such as the brainstem or spinal cord, which may afford less flexibility to the task demands.

**NEW & NOTEWORTHY** An intrinsic hand muscle shows a crossed response to a perturbation of the contralateral index finger. The crossed response is dependent on the direction of the contralateral perturbation but not on the amplitude or the bimanual requirements of the movement, suggesting a far less flexible control policy than those governing crossed responses in more proximal muscles. The crossed response is too fast to be purely mediated by transcortical pathways, suggesting subcortical contributions.

## INTRODUCTION

Everyday movements engage both upper limbs, in many cases to manipulate a common object. To perform these daily actions, the nervous system must coordinate the activity of muscles across both upper limbs together, and this happens in both a predictive and a reactive fashion. An example of a predictive bilateral interaction is seen when one hand is about to act on an object held by the other: in such circumstances, the hand already holding the object shows a predictive increase in grip force, and this anticipatory movement can be adjusted depending on bimanual requirements of the task (Blakemore et al. 1998; Witney and Wolpert 2003).

Bilateral interactions also need to adapt in a fast and reactive fashion to unpredictable perturbations, as illustrated by quotidian tasks such as keeping a tray leveled with both hands. The unexpected addition of an extra load (the “perturbation”) will destabilize the tray, which needs to be rapidly accounted for by both arms in a cooperative fashion: adding an extra load on the middle of the tray will require a different response than when the load is added slightly off center (Bracewell et al. 2003; Dimitriou et al. 2012). Even when a perturbation displaces only one arm, responses can be seen in the unperturbed arm at latencies that are too fast to be purely voluntary (Marsden et al. 1981; Mutha and Sainburg 2009). Responses in the unperturbed arm are typically termed crossed responses (CRs). Several studies have now reliably demonstrated that the amplitude of the CRs to mechanical perturbations flexibly adapts to the bimanual requirements of the task (Diedrichsen 2007; Mutha and Sainburg 2009; Omrani et al. 2013).

This adaptability is compatible with how muscles respond following a mechanical perturbation and is associated with different neural structures. The fastest or short latency response (SLR) in muscles is mediated by spinal pathways, primarily driven by activation of group Ia afferents (Lundberg and Winsbury 1960). The SLR shows limited flexibility to the requirements of the context in which the movement is performed, its amplitude being primarily determined by the size of the perturbation and the background level of muscle activity. The SLR is followed by the medium or long latency responses (collectively termed LLRs), which typically occur at latencies too fast to be under volitional control; accumulating evidence in both animal and human studies strongly suggest that the primary motor cortex (M1) contributes to the LLR, particularly for forearm and hand muscles (Capaday et al. 1991; Cheney and Fetz 1984; Day et al. 1991; Evarts and Tanji 1976; Omrani et al. 2014; Pruszynski et al. 2011). This transcortical contribution to the LLR is thought to underlie its much higher flexibility, whereby its amplitude is sensitive to different aspects of the behavioral task, its goals, as well as the limb biomechanics (Ahmadi-Pajouh et al. 2012; Evarts and Granit 1976; Hammond 1956; Manning et al. 2012; Nashed et al. 2012; Pruszynski et al. 2008; Pruszynski and Scott 2012; Shemmell et al. 2010). In bimanual studies that have recorded from muscles bilaterally, the onset latency of CRs in proximal muscles suggests the mediation of a transcallosal LLR (Dimitriou et al. 2012; Mutha and Sainburg 2009).

The ability of intrinsic hand muscles to participate in CRs remains however unclear. Most previous studies on bimanual control used proximal arm movements during which the involvement of hand muscles was not relevant. Studies that did employ manipulative tasks involving the hands did not directly investigated the response of intrinsic hand muscles (Bracewell et al. 2003; Ohki et al. 2002; White et al. 2008; Witney and Wolpert 2003). As the control of the fingers relies on the activity of both extrinsic and intrinsic hand muscles, it is possible that the bimanual coupling between the fingers reported in these studies could be primarily mediated through activity in forearm muscles.

Do intrinsic hand muscles show CRs? If so, how sensitive are these CRs to perturbation parameters such as direction, magnitude, or bimanual demands of the task? Intrinsic hand muscles show strong LLR responses to a mechanical perturbation (Macefield et al. 1996), so the expectation is that they should show comparable flexibility as seen in more proximal muscles. However, during bimanual finger grip control, CRs show a fast (<70 ms) and inflexible degree of coupling that persists even when handling separate virtual objects with each hand (White et al. 2008), suggesting the underlying contribution of subcortical systems.

To address these two fundamental questions, we investigated CRs in a bimanual task involving isolated finger movements. Our results show that CRs are present in intrinsic hand muscles and they are sensitive to the direction of mechanical perturbations. However, they show reduced sensitivity to the magnitude of the perturbations and the bimanual requirements of the task. Their onset latencies further support the involvement of subcortical structures.

## METHODS

Experiments were performed on a total of 26 human volunteers (20–55 yr old, 17 women, 3 left-handed) who provided informed consent and were reimbursed for their time. Twelve participants took part in *experiments 1* and *2*, and 14 participants took part in *experiment 3*. Not all volunteers participated in all experiments. The sample size was not chosen with consideration of adequate power to detect a prespecified effect. This work was approved by the local ethics committee at Newcastle University (Newcastle upon Tyne, UK).

### Behavioral Task

Participants were asked to control the position of a cursor on the screen using force and position signals from two manipulanda controlled by the left and right index fingers respectively. The left manipulandum consisted of a fixed lever (stainless steel, 5-mm thick) instrumented with strain gauges (RS Components, 5-mm Wire Lead Strain Gauge, stock no. 632-180) to detect applied force. A miniature load cell amplifier (ICA2H, Applied Measurements, Ltd., Aldermaston, Reading, UK) was used in a half-bridge configuration to amplify the force signals from the lever. The right finger manipulandum consisted of a movable lever attached to a motor (Maxon DC motor 218010 with an Escon 50/5 controller) and a potentiometer to detect angular position of the lever. The motor was used to provide a constant load against which to contract and to provide a torque perturbation on some trials. In some sessions, an accelerometer (MEMS accelerometer, MMA7341LC, Freescale Semiconductor) was also attached to the lever to verify the onset latency of movements and perturbations. Participants were seated comfortably with both hands resting on the table with elbows bent at approximately right angles. Moveable support struts were individually placed for each subject to accommodate the varying hand sizes and ensure that hand posture did not drift during the task.

**Fig. 1.**
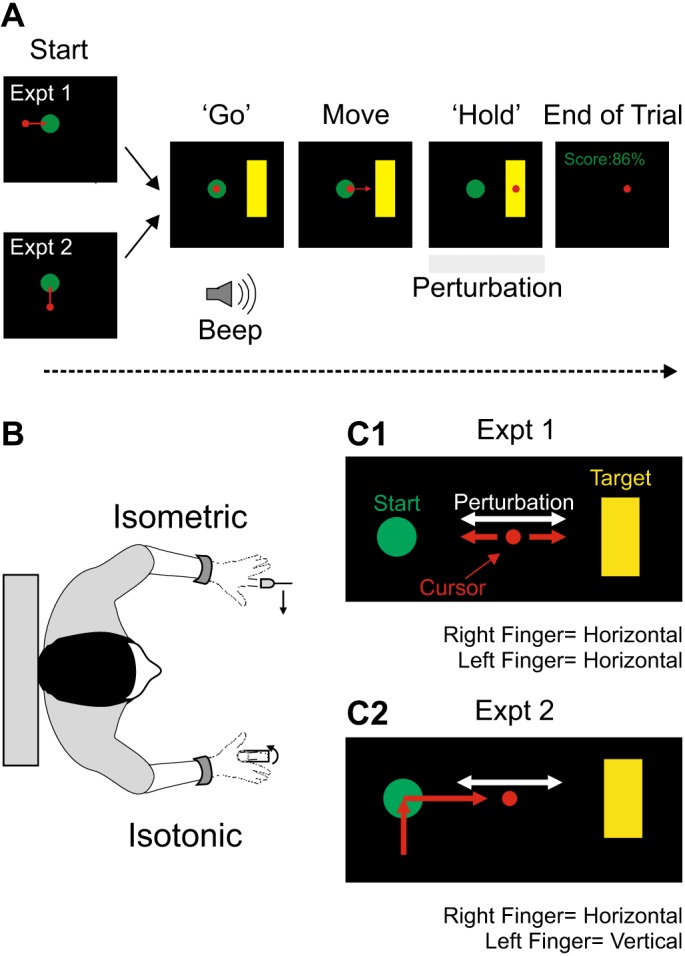
Behavioral task. *A*: trial temporal structure. Participants initiated a trial by moving the cursor inside the start position. After a variable delay (0.5 to 1 s), a “GO” cue instructed the participants to move the cursor into target. The GO cue consisted of the concurrent presentation of the target and a 400-ms beep (2-kHz tone). Participants had then 1 s to reach the target. Once in target, they had to hold the cursor within target for 1 s until the end of the trial, but a perturbation given to the right index finger could eject the cursor from the target. Participants were given a score on what fraction of the hold period they were able to keep the cursor within target. *B*: schematic of the hand arrangement. The left index finger was placed in a fixed lever instrumented with strain gauges. The right index finger was placed in a moveable lever attached to a motor. Participants faced a visual display unit at ~40 cm. The display consisted of a cursor (red) that had to be moved from a starting position (green circle) to a target position (yellow rectangle). *B*: schematic of the two different experimental paradigms using this task. *C1*, *experiment 1*: the cursor could only move along the horizontal axis; its cartesian position was the average of the force and angular position signals from the left and right levers (*Linked* control). *C2*, *experiment 2*: the cursor could move along the horizontal and vertical axes. The force signal from the left lever controlled the cursor position along the vertical direction; the angular position from the right lever controlled the cursor position along the horizontal direction (*Orthogonal* control).

The task was self-paced: participants initiated a trial by moving the cursor into the starting position (indicated by a green circle, see Fig. 1*A*). Note that this always required abduction movements from both fingers (see Fig. 1). Following a random delay (uniform distribution, 0.5–1 s), a “GO” signal consisting of a brief beep (tone at 2 kHz for 400 ms) was concurrently delivered with the appearance of the target (indicated by a vertical yellow rectangle). Participants had 1 s to move the cursor into target from the onset of the GO cue. Once in target, they had to hold the cursor within target for 1 s until the end of the trial, which was signified by the disappearance of the target. The aim was to keep the cursor within target for the whole duration of the hold period (1 s). The width of the cursor was ~2 cm, which corresponded to ~10% of the distance that the cursor had to move on the screen (from Start to Target, Fig. 1*B*).

At the end of each trial, participants were given a feedback score (0–100) indicating how well they did on that trial: this feedback was the percentage of the hold period duration in which the cursor was kept in target (see Fig. 1*A*). Target level of force and target position were set individually for each participant; they were asked to make comfortable isometric abduction contractions with the left index finger and isotonic abduction movements with the right index finger (see Fig. 1*B*) that were approximately midway between its maximal abducted and adducted positions. The left force and right position signals were then used to determine the position of the cursor on the screen, but this varied between experiments (see below). Participants were initially allowed 10–50 trials with no perturbations to get acquainted with the task. To minimize the chance of fatigue, participants were asked to take a 5-min break every ~150 trials. To ensure that hand posture did not change across epochs, marks on the task and hands of the subject were made between breaks.

The task was controlled by a PC running a custom-made program written in Borland Delphi 7. The signals used in the task (force and position signals) were passed into the task program via a National Instruments Data Acquisition system (NIDAQ, USB-6001), sampled at 5 kHz. The same NIDAQ interface was used to output all relevant task-related variables to the capture system: these included six-bit digital markers for the different task events, cursor position, as well as a perturbation signal on every trial.

We performed three experiments. In *experiment 1*, the aim was to test whether CRs in the left first dorsal interosseus (L1DI) were sensitive to the nature of the mechanical perturbation (direction and amplitude) on the contralateral right finger. In *experiment 2*, the aim was to test whether L1DI CRs were sensitive to the bimanual requirements of the task. In both *experiments 1* and *2*, the mechanical perturbation to the right index finger was set up to activate sensory afferents from the right 1DI (R1DI), but muscle afferents from forearm muscles with tendinous insertions onto the index finger were also likely to respond. Therefore, in *experiment 3*, we delivered nerve stimulation at the wrist to determine whether CRs could be elicited through activation of afferents distal to the wrist. Each experiment is described in more detail below.

#### Experiment 1A: sensitivity of CRs to perturbations of different directions.

The aim of this experiment was to examine whether mechanical perturbations of different directions (*Clockwise*-*Anticlockwise*, see Perturbations in Fig. 1) to the right index finger can elicit and modulate CRs on the contralateral L1DI during a bimanual task. Participants were asked to move and hold the cursor within target and achieve the highest score possible per trial. In the instance of perturbations, participants were instructed to return the cursor within target as fast as possible. In this experiment, the cursor could only be moved along the horizontal axis, its position being the average of the signals from the left and right manipulanda. As both fingers contributed to cursor movement along the task relevant dimension (*Linked* control, Fig. 1*C1*), a perturbation could be compensated by either or both fingers. If participants applied too little (<90%) or too much (>110%) force relative to the target value with the left finger, feedback on the screen was presented at the end of each trial, stating that force level was too weak or too strong with the left finger. These trials were not excluded from the data analysis.

#### Experiment 1B: sensitivity of CRs to perturbations of different magnitudes.

The aim of this experiment was to examine whether L1DI CRs are modulated by the amplitude of perturbations delivered to the contralateral right index finger. In this experiment, perturbations to the right index finger were either *Weak* (0.6-Nm torque step) or *Strong* (1 Nm torque step), but the task was otherwise identical to *experiment 1A*. The sequence of *Weak* and *Strong* perturbation was randomized.

#### Experiment 2: sensitivity of CRs to bimanual demands of the task.

The aim of this experiment was to determine whether the bimanual requirement of the task affects CRs in the left unperturbed index finger. In contrast to *experiment 1*, where both fingers controlled the position of the cursor in the same axis (*Linked* control, Fig. 1*C1*), in this experiment the cursor moved in both axes (*Orthogonal* control, Fig. 1*C2*). Each finger controlled a single axis: the right index finger controlled the horizontal axis and the left index finger controlled the vertical axis. Under these circumstances, a perturbation of the right finger only disturbed the cursor along the horizontal axis controlled by the right finger. Therefore, the left and unperturbed finger could not compensate for the perturbation in any task-relevant way. If CRs are sensitive to the bimanual requirements of the task, CRs in the unperturbed left index finger during *Orthogonal* control are expected to differ from those during *Linked* control (*experiment 1A*). The amplitude of the perturbations was the same as *experiment 1A*.

#### Experiment 3: CRs to distal sensory stimulation.

A mechanical finger perturbation may activate spindle afferents from several muscles, both intrinsic and extrinsic to the hand. The aim of this experiment was to determine whether isolated activation of distal hand muscles could elicit CRs. With the use of a similar task as in *experiment 1A*, a different cohort of 14 participants was instructed to move and hold a cursor within target, and while in target, weak peripheral nerve stimulation of the median and ulnar nerves was delivered at the wrist. The aim was to examine whether the reafference from electrically elicited weak twitches in the intrinsic hand muscles could evoke CRs. A distal cathode was used to ensure the direct orthodromic motor activation of the intrinsic hand muscles, while minimizing orthodromic sensory conduction and antidromic motor excitation of forearm muscles with anodal block (block effectiveness was not tested). To compare the latencies of CRs to electrical and mechanical perturbations, the same participants were asked to perform a separate batch of trials (*n* = 50) in which mechanical perturbations were delivered to the right finger as in *experiment 1A*.

### Data Capture

Data was captured using a micro1401 (Cambridge Electronic Design, Cambridge, UK) with an expansion top box (giving a total of 16 waveform capture channels). All waveform channels were sampled at 5 kHz. The 1401 controlled the torque level on the motor through a DAC channel providing a command signal to the motor encoder. A sequencer script running on the 1401 was responsible for delivering the perturbations.

Muscle activity (EMG) was recorded through disposable snap electrodes (1440-25, Natus) placed over the belly and a distal location from the muscle of interest. During the perturbation experiments (*experiments 1* and *2*), left and right (1DI) were recorded. During the nerve stimulation experiment (*experiment 3*), 1DI and abductor pollicis brevis (AbPB) were recorded. To attenuate movement and mechanical perturbation artefacts, EMG signals were filtered and amplified (band pass filtering at 30 Hz to 2,000 kHz, gain ranging between 1 and 2 K across participants) using a Neurolog system (Digitimer, Welwyn Garden City, UK), which consisted of amplifiers (NL824), isolators (NL820A), and filter modules (NL125), before being captured along with the other task signals. Digital events captured included the time of the perturbation, as well as markers corresponding to the different task phases. The maximal voluntary contraction (MVC) for each muscle was recorded at the end of the experiment.

### Mechanical Perturbations

On most trials (75%), the motor applied a torque perturbation to the right finger either in the *Clockwise* or *Anticlockwise* direction in equal proportions. *Clockwise* perturbations produced a stretch in the right 1DI; *Anticlockwise* perturbations unloaded (reduced the level of contraction) the right 1DI. These perturbations consisted of a step change in motor torque from a baseline level of 0.2 to ± 0.6 Nm (*experiments 1A* and *2*, *Weak*) and ±1 Nm (*experiment 1B*, *Strong*) for a duration of 250 ms, followed by a ~500-ms ramp return to baseline. Perturbations were given at ~200 ms after hold period onset. The order of all perturbation parameters such as direction and amplitude was randomized from trial to trial. Perturbations were given in both directions to minimize predictability and planning for the response.

### Electrical Stimulation

Afferent nerve stimulation of the median and ulnar nerves at the wrist was delivered using a bar electrode (8-mm diameter contacts, 30 mm apart, P10-4z1, MedCaT Supplies, Klazienaveen, The Netherlands) placed at wrist (cathode distal). Median nerve stimulation was delivered approximately between the tendons of flexor carpi ulnaris and the palmaris longus. Ulnar nerve stimulation was delivered ~3 cm from the distal crease at the wrist (Kimura, 2001). A constant current stimulator was used (DS7AH, Digitimer) to deliver a monophasic square pulse (0.5-ms width) at a mean stimulation rate of ~1 Hz (random interstimulus rate of 0.5 to 1.5 Hz) with an intensity of 1.2 to 1.5× motor threshold (MT) for the corresponding nerve. The MT for each nerve was determined by close visual inspection of muscle twitches in response to the stimulation (AbPB for the median nerve and 1DI for the ulnar).

### Analysis

Waveform data were first aligned relative to a behavioral marker or mechanical perturbation/electrical stimulus event and then averaged across trials. In the case of muscle data, the EMG signal was full wave rectified before averaging. For each subject, the data were normalized by dividing by the mean value of the prestimulus epoch (200 ms) to allow comparison and averaging across participants.

Following a mechanical perturbation, muscles respond with multiple, typically two to three, bursts of activity at latencies too fast to be voluntary. Various terminologies have been used to refer to them in the literature. Early work in humans and nonhuman primates used the terms M1, M2, and M3 (Lee and Tatton 1975) or SLR and LLR (Hammond 1955; Matthews 1986; Rothwell et al. 1980), while more recent work has used the terms R1, R2, and R3 (Pruszynski et al. 2008, 2009; Pruszynski et al. 2008). Based on known central and peripheral delays, the earliest component (SLR) is too fast to be mediated by supraspinal pathways. The SLR typically encompasses EMG activity ~20 to 45 ms after the perturbation for shoulder muscles and ~35 to 60 ms for intrinsic hand muscles (Baudry et al. 2009; Macefield et al. 1996). Here, we observed responses as early as 30 ms and consider the period between 30 and 60 ms as the SLR. Intrinsic hand muscle responses beyond 60 ms up until ~110 ms are considered (Baudry et al. 2009; Macefield et al. 1996) long-latency responses (LLRs). Although some studies (Kurtzer et al. 2009; Pruszynski et al. 2009) have subdivided the LLR period into two subepochs (R2 and R3), here we refer to reflex responses between 60 and 110 ms collectively as the LLR period and to responses between 110 and 200 ms as the early voluntary period (V).

Nonstationary prestimulus/perturbation EMG levels can affect the response period. To account for this, we estimated the baseline EMG levels by using the trials with no perturbation and fitting a regression line through the ±200 ms around perturbation onset. The EMG response was first divided by the mean baseline value (estimated from −200 to 0 ms before perturbation onset), and the predicted baseline was then subtracted from the mean response to the perturbation.

The onset latency of EMG responses was taken as the first instance of at least 10 ms that was either larger or smaller than the mean level of a 200-ms prestimulus/perturbation baseline period (Perez et al. 2014). In addition, a continuous 5-ms epoch within the 100-ms postperturbation had to be larger or smaller than the background level of noise (2× standard deviation). Time-resolved significance levels of EMG responses were estimated using sliding *t* tests (the width of the window is specified within the relevant text). EMG responses were compared between perturbation conditions (*Clockwise*-*Anticlockwise*, *Weak*-*Strong*) and control modes (*Linked*-*Orthogonal*) across epochs (SLR, LLR, and V) using two-way mixed ANOVAs and multiple Bonferroni-corrected paired *t* tests (adjusted significance level α = 0.0167). The data met all the assumptions of the tests used. Only in one instance (R1DI responses in *experiment 2*), normality and homogeneity of variances were mildly violated due to an outlier participant. In this case, we excluded the outlier participant from the data set to ensure that both assumptions were met.

## RESULTS

### Experiment 1A: Sensitivity of CRs to Perturbations of Different Directions

This experiment aimed to examine whether mechanical perturbations of different directions to the right index finger can elicit and modulate CRs on the contralateral L1DI during a bimanual task. Thirteen volunteers participated in this experiment, but one participant who did not show significant CRs was excluded (see methods). One of the remaining 12 participants was left-handed, but that participant’s inclusion did not significantly change the results. Participants were able to keep the cursor in target for most of the 1-s hold period during the unperturbed trials (mean score 93% across participants, range of mean participant scores 82–99%). Compared with unperturbed trials, participants fared significantly worse in trials with *Clockwise* (mean: 69%, range: 36–90; paired *t* test, *P* < 0.002) and *Anticlockwis*e (mean: 64%, range: 29–85%; paired *t* test, *P* < 0.002) perturbations.

Relative to MVC, L1DI contraction levels were higher than R1DI levels but not significantly (R1DI: 19%, range: 5–35%; L1DI: 25%, range: 10–30%; paired *t* test, *P* > 0.1). Eight participants performed 600 trials in this task, and the rest performed 300 trials. The target degree of movement of the right index finger was variable across participants, due to individual differences in hand sizes and joint ranges. The average angular displacement between start and target positions was 7° (range 5–12°).

**Fig. 2.**
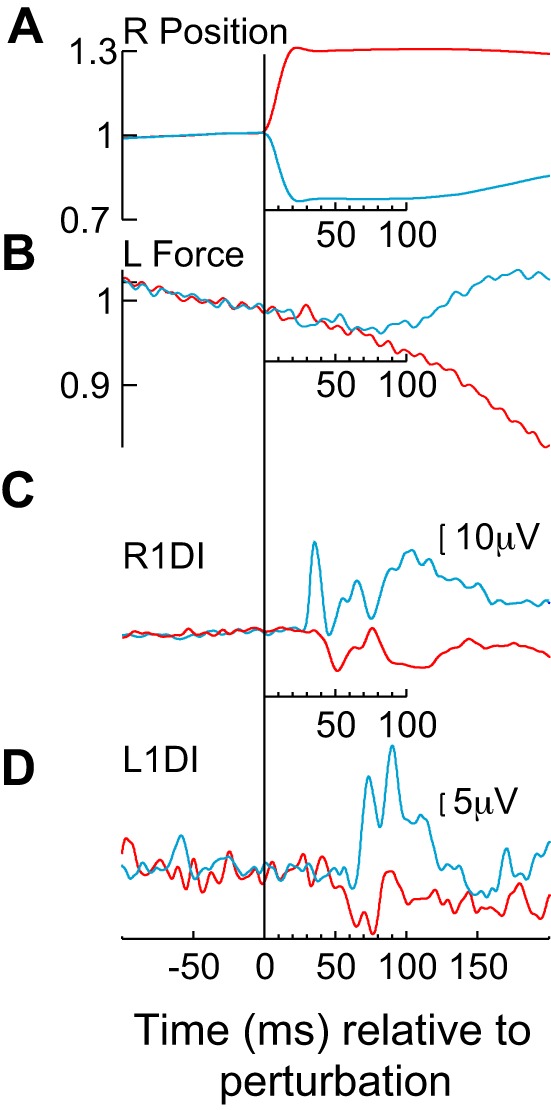
Example responses from a single participant. Mean task and bilateral EMG signals aligned to perturbation onset at an expanded timescale. Signals from trials with *Clockwise* perturbations are in blue, and those from *Anticlockwise* perturbations are in red. *A*: mean position of right finger lever. *B*: mean force signal from left lever. *C*: mean EMG level of right first dorsal interosseus (R1DI). *D*: mean EMG level of left first dorsal interosseus (L1DI).

Figure 2 shows the averaged signals for one example participant aligned to perturbation onset. Traces in blue correspond to trials with *Clockwise* perturbations, while traces in red correspond to trials with *Anticlockwise* perturbations. Figure 2, *A* and *B*, shows angular position and force signals from the right and left levers, respectively. Figure 2, *C* and *D*, shows mean EMG traces from the R1DI and L1DI muscles. Torque perturbations of the right lever elicited responses in both right and left 1DI muscles (Fig. 2, *B* and *C*). Force signals from the left lever (Fig. 2*B*) deviate from each other as early as ~80 ms in this subject, persisting at least until 200 ms. EMG traces from the R1DI muscle (Fig. 2*C*) illustrate characteristic SLR and LLR responses for *Clockwise* perturbations and suppressed muscular activity for *Anticlockwise* perturbations. Figure 2*D* shows clear L1DI CRs to both perturbation directions. The onset of the earliest inhibitory CRs to *Anticlockwise* perturbations (blue) was at 55 ms, while the onset of the earliest facilitatory CRs to *Clockwise* perturbations (red) was at 68 ms.

**Fig. 3.**
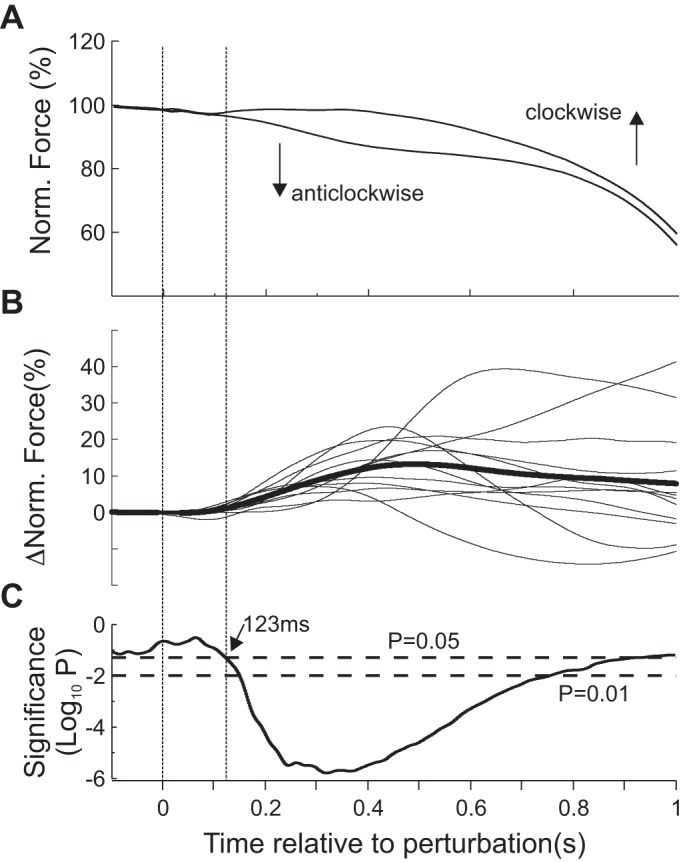
Population responses in left lever force signals during *experiment 1A*. *A*: average force signals from the left lever for 12 participants. Force signals were normalized to the required force that each subject had to exert to move the cursor into target. *B*: difference in force signal from the left lever with perturbation direction for each participant (thin grey lines) and average across all participants (thick black line). *C*: time-resolved (10-ms sliding window) *P* values from pair-wise comparisons (*t* tests) between *Clockwise* and *Anticlockwise* perturbation directions. The *P* value is displayed in logarithmic scale base (10) and is not Bonferroni adjusted. The first time point at which there is a significant difference between the two perturbation directions is 123 ms.

Figure 3 displays force signals from the left lever, normalized to target force value. The average force traces from all participants are displayed in Fig. 3*A*. At ~95 ms the force signals consistently deviate between *Clockwise* and *Anticlockwise* directions. Figure 3*B* shows force signal differences between *Clockwise* and *Anticlockwise* perturbation trials. Note that positive values illustrate larger forces during *Clockwise* perturbations. Figure 3*C* shows the time-resolved *P* values resulting from a 10-ms sliding window paired *t* test between *Clockwise* and *Anticlockwise* perturbation trials (*P* values are expressed in a log10 base). The first instance to show significantly different force CRs to *Clockwise* and *Anticlockwise* perturbations is at 123 ms (*P* < 0.05).

**Fig. 4.**
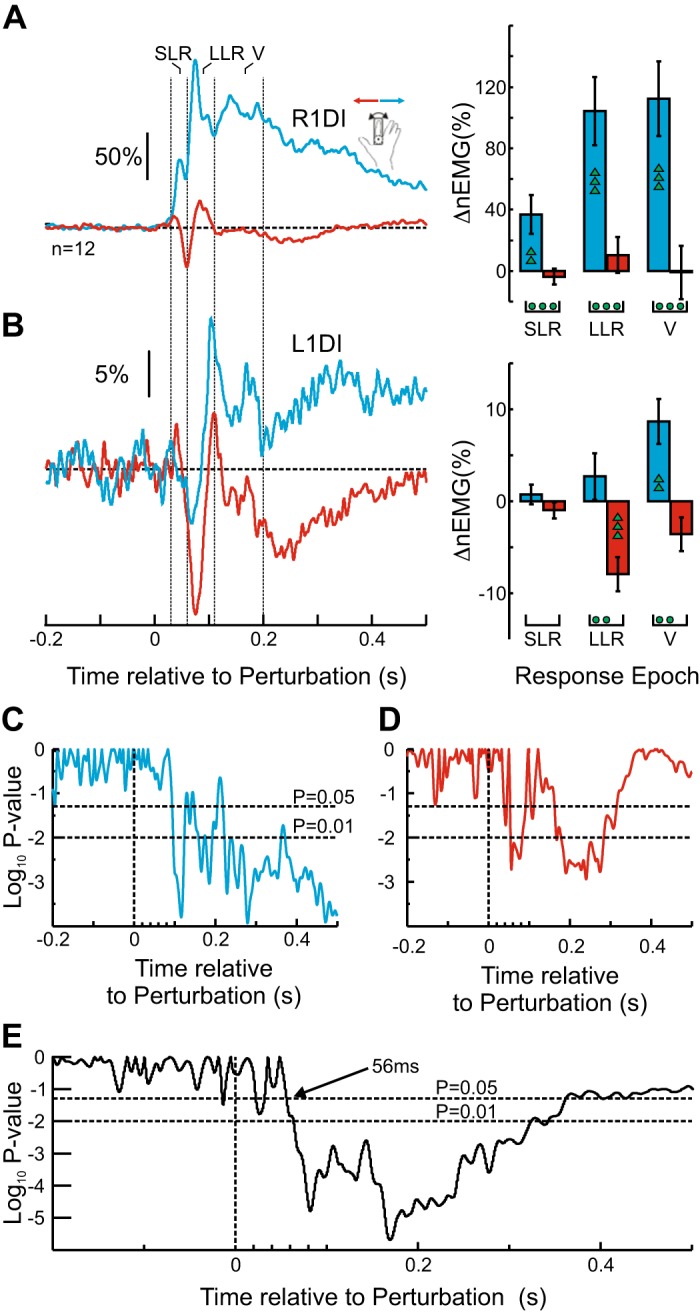
Effect of perturbation direction on crossed responses (CRs). *A*: participant-averaged, normalized EMG (nEMG) recordings from right first dorsal interosseus (R1DI) during *Clockwise* (blue) and *Anticlockwise* (red) perturbations. The same color code applies to plots *B*–*D*: short latency response (SLR), long latency response (LLR), and voluntary period (V) indicate different response epochs (SLR: 30–60 ms; LLR: 60–110 ms; V: 110–200 ms). The EMG was normalized to the mean background level of EMG activity for each participant before averaging. *Right*: R1DI epoch analysis. Each bar shows the mean EMG levels for the corresponding epochs. Error bars show means ± SE. Green triangles indicate significant differences from baseline for each perturbation direction and epoch. Green circles indicate significant differences between perturbation directions. The number of green markers indicates the level of significance (1 marker: *P* < 0.05; 2 markers: *P* < 0.0167; 3 markers: *P* < 0.001). The horizontal black lines in each data column indicate the mean EMG level relative to baseline. *B*: participant-averaged left first dorsal interosseus (L1DI) nEMG during *Clockwise* and *Anticlockwise* perturbations. *Right*: L1DI epoch analysis. *C*: rime-resolved *P* values from sliding paired *t* tests contrasting L1DI CRs and EMG baseline for *Clockwise* perturbations. The *P* value is displayed in logarithmic scale base (10). *D*: same as *C* but for *Anticlockwise* perturbations. *E*: time-resolved (10-ms sliding window) *P* values from pair-wise comparisons (*t* tests) between L1DI CRs to *Clockwise* and *Anticlockwise* perturbation directions. The first time point at which there is a significant (*P* = 0.05) difference between the two perturbation directions is at ~56 ms. Note that for *C*, *D*, and *E* the *P* values are not Bonferroni adjusted.

Figure 4 displays 1DI muscle responses across all participants. Figure 4, *A* and *B*, *left*, shows the mean R1DI and L1DI responses to *Clockwise* (blue) and *Anticlockwise* (red) perturbation directions. Figure 4, *A* and *B*, *right*, shows the mean EMG values during the three epochs of interest (SLR: 30–60 ms; LLR: 60–110 ms; V: 110–200 ms). The green circles show which epochs showed a significant difference (paired *t* test) between perturbation directions; the green triangles show which responses were significantly different from baseline values. A single green marker indicates a *P* < 0.05, two are for a *P* < 0.0167 (Bonferroni-corrected significance level), and three are for *P* < 0.001. R1DI responses showed the expected short and long latency components to a direct mechanical perturbation. A two-way mixed ANOVA for R1DI responses revealed a significant effect of epoch (*F* = 5.47, df = 2, *P* < 0.01) and direction of perturbation (*F* = 28.36, df = 1, *P* < 0.0002). There was no significant interaction between the two (epoch × direction, *F* = 2.63, df = 2, *P* < 0.09). In response to *Clockwise* perturbations, which caused R1DI stretching, there was a highly significant response in all epochs (SLR: 36.7%; LLR: 103.4%; V: 110.9% relative to preperturbation EMG level, all *t* test *P* < 0.001), while responses to *Anticlockwise* perturbations were generally much smaller with some being inhibitory (SLR: −8.3; LLR: 15.1%; V: −1.4%). R1DI responses were significantly different between the two perturbation directions (*P* < 0.001, paired *t* test) in all epochs. The *Clockwise* perturbation elicited an R1DI SLR in all participants.

*Clockwise* and *Anticlockwise* perturbations also elicited clear L1DI CRs (Fig. 4*B*). A two-way mixed ANOVA revealed a significant effect of epoch (*F* = 6.2, df = 2, *P* < 0.007), and direction of perturbation (*F* = 18.2, df = 1, *P* < 0.0011), as well as for the interaction between the two (epoch × direction, df = 2, *F* = 7.71, *P* < 0.0026), which indicates that the time course of CRs was different for the two perturbation directions. *Clockwise* perturbations elicited a weak early inhibition that peaked at ~68 ms, followed by a larger facilitation at longer delays (LLR: 2.7; V: 8.7%). *Anticlockwise* perturbations elicited late inhibition during the long latency epoch (LLR: −8%; V: −3.6%). L1D1 CRs to *Clockwise* and *Anticlockwise* perturbations were significantly different in all epochs except SLR (Fig. 4*B*. *P* < 0.0167).

Figure 4, *C* and *D*, shows the time-resolved significance of L1DI CRs relative to the preperturbation period. Figure 4*C* shows the *P* values from a sliding paired *t* test (5-ms wide window) of CRs to *Clockwise* perturbations and Fig. 4*D* to *Anticlockwise* perturbations. These plots support the epoch analysis shown in Fig. 4*B*, with CRs to *Anticlockwise* perturbations showing a considerably earlier onset than CRs to *Clockwise* perturbations. Figure 4*E* shows the time-resolved *P* values from a sliding paired *t* test (5-ms wide window) of the difference between L1DI CRs to *Anticlockwise* and *Clockwise* perturbation directions. Although there is a brief period of significance (*P* < 0.05), which likely corresponds to the short latency facilitation seen in CRs to *Anticlockwise* perturbations (red trace in Fig. 4*B*, SLR), a long-lasting difference between CRs to both perturbations directions starts at 56 ms.

Altogether, group EMG analysis show CRs in the unperturbed L1DI muscle that are sensitive to the direction of the perturbation in the contralateral right index finger. Interestingly, differences between CRs to different perturbation directions begin as early as 56 ms, suggesting the involvement of pathways under reduced volitional control.

### Experiment 1B: CRs to Perturbations of Different Magnitudes

To further characterize the behavior of the CRs observed in *experiment 1A*, we investigated whether they modulate with perturbations of different magnitude. One week after participating in *experiment 1A*, 7 of the previous 12 volunteers participated in *experiment 1B*. Except for some perturbations being stronger on some trials (see methods), the task was otherwise identical to *experiment 1A*. Performance during unperturbed trials (mean score = 92%) was comparable to performance during unperturbed trials in *experiment 1A* (paired *t* test, *P* > 0.1). Also consistent with performance in *experiment 1A*, participants fared significantly worse in perturbation trials (mean score for perturbation trials = 69%, paired *t* test, *P* < 0.01). Trial scores were significantly lower (paired *t* test, *P* < 0.01) for *Strong* perturbations (mean scores of 62 vs. 76% for trials with *Strong* and *Weak* perturbations respectively). As *experiment 1A* showed different CRs to *Clockwise* and *Anticlockwise* perturbations, we performed the remaining analysis separately for each perturbation direction.

**Fig. 5.**
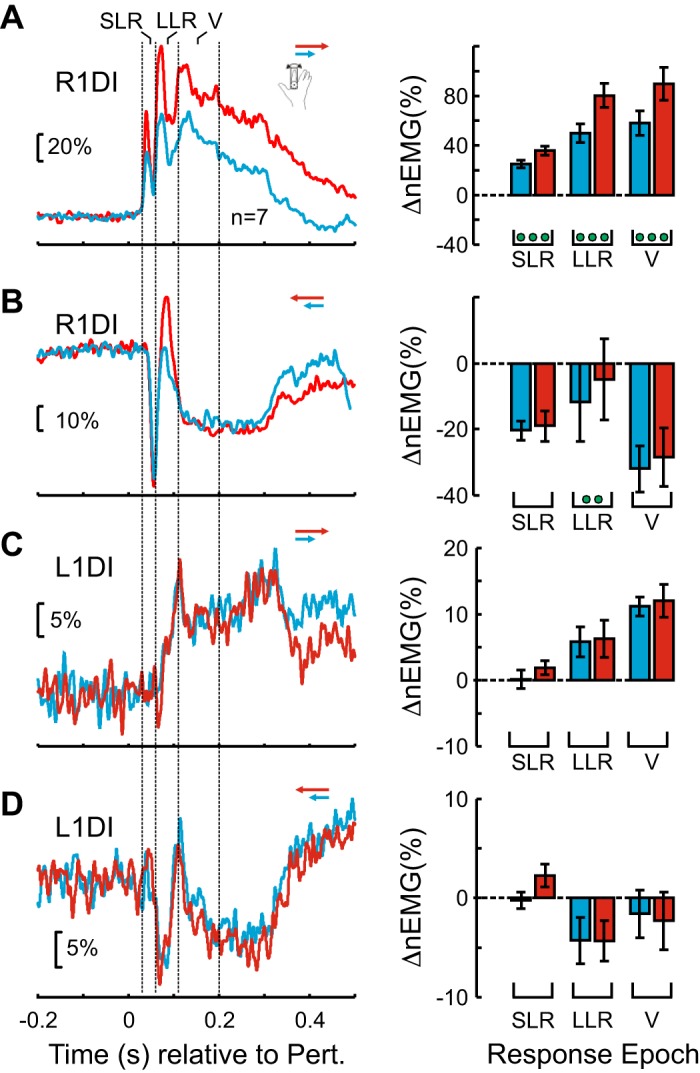
Effect of perturbation amplitude on crossed responses (CRs). *A*: right first dorsal interosseus (R1DI) responses to *Weak* (blue) and *Strong* (red) *Clockwise* perturbations. The same color code applies to the rest of the figure. *Left*: participant-averaged, normalized R1DI EMG (nEMG) recordings. Short latency response (SLR), long latency response (LLR), and voluntary period (V) indicate different response epochs (SLR: 30–60 ms; LLR: 60–110 ms; V: 110–200 ms). The EMG was normalized to the mean background level of EMG activity for each participant before averaging. *Right*: epoch analysis where each bar represents the mean EMG levels for the corresponding epochs. Error bars show means ± SE. The green circles indicate significant differences (paired *t* test) between *Weak* and *Strong* perturbations. The number of green markers indicates the level of significance (2 markers: *P* < 0.0167; 3 markers: *P* < 0.001). *B*: same as *A* but for *Anticlockwise* perturbations. *C*: same as *A* but for L1DI CRs to *Clockwise* perturbations. *D*: same as in *C* but for L1DI CRs to *Anticlockwise* perturbations. No significant differences were found in any of the L1DI epochs tested.

Figure 5 summarizes the EMG response results. Figure 5, *left*, shows the average EMG responses, with red traces corresponding to responses to *Strong* perturbations and blue traces corresponding to *Weak* perturbations. Figure 5, *right*, the mean EMG levels for the corresponding epochs (as in Fig. 4), with green circles indicating the significance level (two circles, *P* < 0.0167; three circles, *P* < 0.001).

Separate two-way mixed ANOVAs (perturbation magnitude, response epoch) for each muscle and perturbation direction revealed highly significant effects of perturbation magnitude (*F* = 50.1, df = 1, *P* = 0.0002) on R1DI responses to *Clockwise* perturbations (Fig. 5*A*). *Strong* perturbations resulted in significantly larger responses across epochs (paired *t* test, *P* < 0.001), especially in LLR and V (significant interaction between epoch and perturbation amplitude; *F* = 19.5, df = 2, *P* = 0.0001). However, the magnitude of *Anticlockwise* perturbations only affected R1DI LLR responses (significant interaction between epoch and perturbation magnitude, *F* = 4.13, df = 2, *P* = 0.0389; LLR paired *t* test, *P* < 0.0017).

In contrast, the magnitude of the perturbations did not modulate the amplitude of L1DI CRs in any perturbation direction (Fig. 5*C*, *Clockwise*: *F* = 0.51, df = 1, *P* = 0.49; Fig. 5*D*, *Anticlockwise*: *F* = 0.33, df = 1,*P* = 0.58) nor was there an interaction between epoch and perturbation magnitude (*Clockwise*: *F* = 0.38, df = 2, *P* = 0.69; *Anticlockwise*: *F* = 3.19, df = 2, *P* = 0.07).

These results suggest that the magnitude of the perturbations did not have an impact on the amplitude of the CRs in the unperturbed L1DI muscle.

### Experiment 2: Sensitivity of CRs to Bimanual Demands of the Task

With this experiment, we examined whether CRs were sensitive to the bimanual requirements of the task (see methods). In contrast to *experiment 1* (in which both hands controlled the same cursor along the same axis, *Linked* control, Fig. 1*C1*), in *experiment 2* each hand controlled the cursor movement along different orthogonal axes (*Orthogonal* control, see Fig. 1*C2*). Seven of the same participants in *experiment 1A* participated in *experiment 2*; the order was randomized. The participants performed similarly in unperturbed trials during *Orthogonal* and *Linked* control (mean score = 87%, paired *t* test, *P* > 0.05). However, they performed significantly worse in perturbed trials during *Orthogonal* control (mean score: 43%, range: 10–76%, paired *t* test, *P* < 0.001). Note that in *Orthogonal* control, the cursor position in the perturbed horizontal axis was exclusively determined by the position of the right lever (Fig. 1*C2*); therefore, the same mechanical perturbation introduced lager deviations along the horizontal axis in *Orthogonal* control than in *Linked* control. In comparing the results between *experiments 1A* and *2*, it is important to ensure that the perturbations were comparable. Paired *t* tests did not reveal significant differences between the peak velocities of the right lever or their time of occurrence in *experiments 1A* and *2* (*P* > 0.1).

**Fig. 6.**
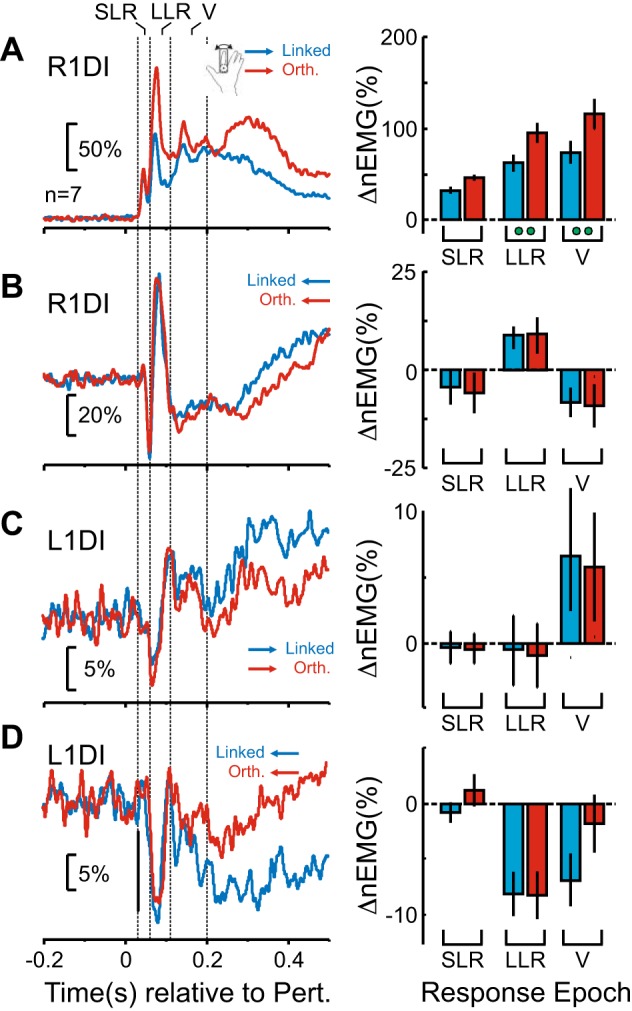
Effect of bimanual task requirements on crossed responses (CRs). *A*: right first dorsal interosseus (R1DI) responses to *Clockwise* perturbations during the *Linked* control (blue) and *Orthogonal* control (red). The same color code applies to the rest of the figure. *Left*: participant-averaged, normalized R1DI EMG (nEMG) recordings. Short latency response (SLR), long latency response (LLR), and voluntary period (V) indicate different response epochs (SLR: 30–60 ms; LLR: 60–110 ms; V: 110–200 ms). The EMG was normalized to the mean background level of EMG activity for each participant before averaging. *Right*: R1DI epoch analysis. Each bar shows the mean EMG levels for the corresponding epochs. Error bars show means ± SE. The green circles indicate significant differences (paired *t* test) between *Linked* and *Orthogonal* control. The number of green markers indicates the level of significance (1 marker: *P* < 0.05; 2 markers: *P* < 0.0167). *B*: same as in *A* but for *Anticlockwise* perturbations. *C*: L1DI CRs to *Clockwise* perturbations during *Linked* control and *Orthogonal* control. *D*: same as *C* but for *Anticlockwise* perturbations.

Figure 6 summarizes the EMG results. Figure 6, *left*, shows the average EMG responses, with red traces corresponding to responses to perturbations during *Orthogonal* control and blue traces during *Linked* control. Figure 6, *right*, shows the mean EMG levels for the corresponding epochs, with green circles indicating the significance level (two circles, *P* < 0.0167). A two-way mixed ANOVA (response epoch, bimanual demands) for each muscle and perturbation direction revealed a significant effect of bimanual demands of the task (*F* = 20.44, df = 1, *P* = 0.003), as well as a significant interaction between epoch and bimanual demands (*F* = 10.58, df = 2, *P* = 0.001) in R1DI responses to *Clockwise* perturbations. Paired *t* tests showed that these R1DI responses to *Clockwise* perturbations were significantly larger in the LLR (*P* = 0.0023, paired *t* test) and V (*P* = 0.0039, paired *t* test) epochs during *Orthogonal* control (Fig. 6*A*). However, no effects of bimanual task demands were observed in R1DI responses to *Anticlockwise* perturbations (Fig. 6*B*), either in terms of task context alone (*F* = 2.97, df = 1, *P* = 0.13) or in the interaction of epoch with task demands (*F* = 1.77, df = 2, *P* = 0.21).

A similar picture emerged for L1DI CRs, where the bimanual task demands were not significant in either *Clockwise* (Fig. 6*C*, *F* = 0.01, df = 1, *P* = 0.92) or *Anticlockwise* (Fig. 6*D*, *F* = 0.61, df = 1, *P* = 0.46) perturbation directions. There was also no significant interaction between bimanual task demands and epoch for *Clockwise* (Fig. 6*C*, *F* = 0.02, df = 2, *P* = 0.97) or *Anticlockwise* (Fig. 6*D*, *F* = 85, df = 2, *P* = 0.45) directions.

These results suggest that only muscle responses from the perturbed finger modulated with the *Linked* and *Orthogonal* requirements of the bimanual task.

### Experiment 3: CRs to Activation of Distal Afferents

Muscle afferents from both intrinsic and extrinsic muscles of the hand could mediate CRs to mechanical perturbations in a bimanual finger task. To better characterize CRs elicited by intrinsic muscle afferents of the contralateral finger, we investigated CRs following electrical stimulation at the wrist in a separate cohort of 14 participants (see methods). Two of the participants in this cohort were left-handed, but their inclusion did not significantly change the results.

**Fig. 7.**
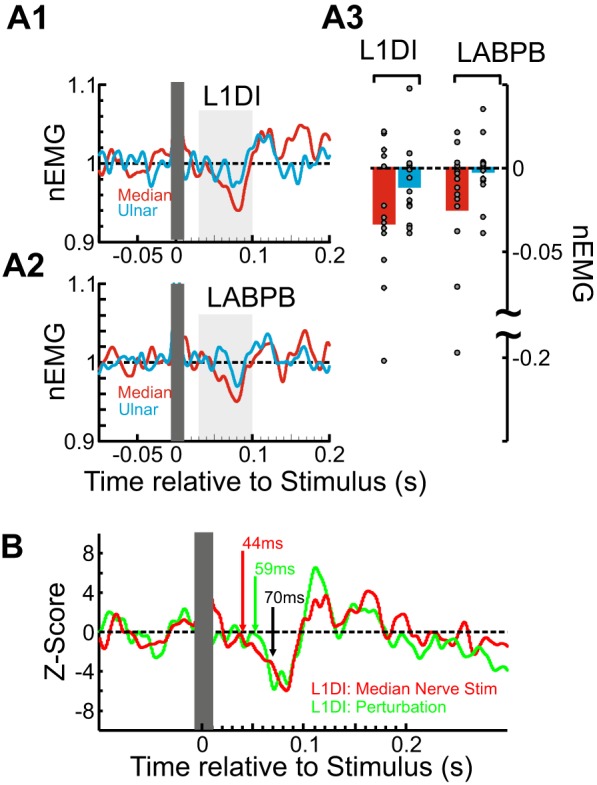
Crossed responses (CRs) from afferents distal to the wrist. *A*: participant-averaged left first dorsal interosseus (L1DI; *A1*) and left abductor pollicis brevis (LABPB; *A2*) CRs to single shock stimulation of the contralateral median (red) and ulnar (blue) nerves at the right wrist (1.2–1.5× motor threshold). The vertical gray boxes cover the stimulus artifact. For each participant, CRs were normalized to the mean background level of EMG (nEMG) before averaging. In both muscles there is a clear response to contralateral nerve stimulation. *A3*: bar plot showing the mean CR amplitude (30–100 ms, light gray box in *A1* and *A2*) to each nerve stimulation at each muscle. Same color code as in *A1* and *A2*. Individual values for each participant are overlaid. Note the larger suppression from contralateral median nerve stimulation. *B*: participant-averaged Z-scored L1DI CRs to right median nerve stimulation (red) and to *Anticlockwise* perturbations of right index finger (green). The colored arrows indicate the inhibitory CR onset latencies (44 and 59 ms). The black arrow indicates the minimum expected delay (70 ms) for a transcallosal pathway.

We recorded responses in the L1DI and LAbPB to electrical stimulation of the right median and ulnar nerves at the wrist at suprathreshold intensities (1.2–1.5× MT, no. of stimuli = 300). The results are summarized in Fig. 7. CRs were normalized relative to the prestimulus background (Fig. 7, *A1* and *A2*; see methods). We observed a clear suppression in both L1DI and LABPB muscles following stimulation of contralateral sensory afferents at delays of ~50 to 100 ms. In the L1DI, we observed a mean 3.6% suppression to median nerve stimulation and a weaker 1.2% suppression to ulnar nerve stimulation. Comparable values were observed in AbPB (suppression of 2.8 vs. 0.1% for median and ulnar nerves respectively). Fig. 7*A3* shows the individual mean CRs values for each muscle and nerve stimulated. Median nerve stimulation produced a nonsignificantly stronger suppression in EMG activity (red bars in Fig. 7*A3*) compared with stimulation of the ulnar nerve (blue bars in Fig. 7*A3*). When combined across both muscles, however, the difference was significant (paired *t* test of responses to ulnar vs. median nerve stimulation with responses combined across both muscles, *P* < 0.02).

To compare the latencies of CRs to electrical and mechanical perturbations, we also gave a small number of perturbations to the index finger (*n* = 50; same perturbation parameters as in *experiment 1A*) while the same participants performed an isometric contraction with the left index finger (see methods). Figure 7*B* overlays the mean CRs to electrical (red: median nerve) and mechanical perturbations (green: *Anticlockwise*) that displayed stronger suppression. CRs were Z-scored to compare their temporal profile. CRs to both electrical and mechanical perturbations exhibited suppression at similar onset latencies, 44 and 59 ms, respectively.

These results suggest that activation of afferents distal to the wrist can produce CRs in a contralateral intrinsic hand muscle. CRs to both electrical and mechanical perturbations display suppression relative to background EMG levels at comparable onset latencies.

### Analysis of CR Onset Latencies

The onset latencies of CRs observed in the unperturbed 1DI were consistently faster than ~110 ms; therefore, it seems unlikely for them to be under volitional control. A more detailed analysis of the distribution of CR latencies could shed light into the underlying neural pathways mediating them. To this end, we examined the onset latencies of R1DI responses and L1DI CRs (see *Analysis* in methods) to *Clockwise* and *Anticlockwise* perturbations measured in *experiment 1A*.

Fast responses in perturbed muscles (typically 30–60 ms for an intrinsic hand muscle, SLR) are mainly mediated by spinal pathways, while longer latency responses (>60 ms, LLR) may be mediated through multiple routes including the cortex (Macefield et al. 1996). CRs may require additional delays for crossing over the midline. Therefore, considering that 10 ms is the shortest reported delay for transcallosal inhibition in humans (Ferbert et al. 1992), CRs with onset latencies less than ~70 ms might be too fast to be mediated via transcallosal pathways.

**Fig. 8.**
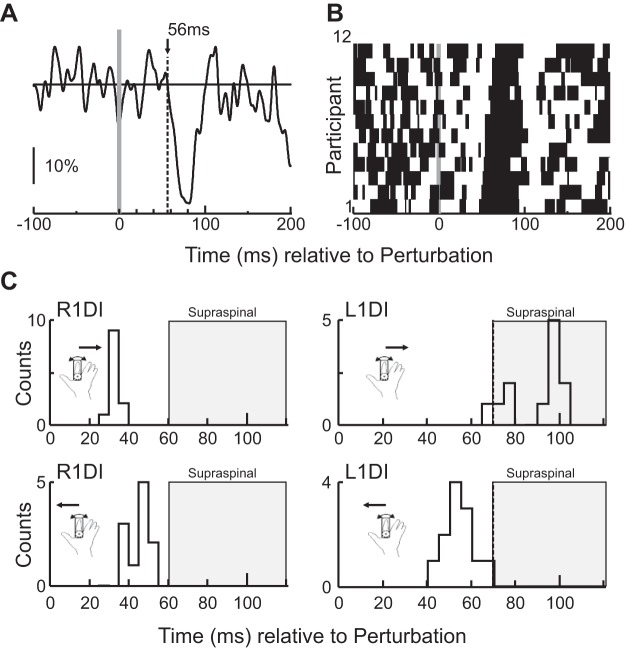
Crossed response (CR) onset latencies. *A*: left first dorsal interosseus (L1DI) EMG CRs from 1 participant. *B*: responses aligned to perturbation onset for each participant. Bins below the background level of EMG are in black, and bins above the background level of EMG are in white. *C*: distribution of onset latencies. *Left*: right first dorsal interosseus (R1DI) CR latencies to *Clockwise* (*top*) and *Anticlockwise* (*bottom*) perturbations. *Right*: L1DI CR latencies to *Clockwise* (*top*) and *Anticlockwise* (*bottom*) perturbations. Gray boxes indicate delays which may suggest the involvement of supraspinal structures. Note that for the L1DI, the boxes start at 70 ms, which is the expected delay for the fastest transcallosal CRs.

Figure 8*A* shows an example CR from one of the participants in *experiment 1A*, while Fig. 8*B* shows the distribution (for each participant) of bins with values above (white) and below (black) the baseline. Figure 8*C* shows the latency distribution histograms for the different muscles and perturbation directions. R1DI response mean latency to *Clockwise* and *Anticlockwise* perturbations (Fig. 8*C*, *left*) was 33.4 and 45.4 ms respectively, with a pairwise difference of 12 ms. For an intrinsic hand muscle, both latencies are suggestive of a spinal pathway. The corresponding latencies for L1DI CRs (Fig. 8*C*, *right*) were considerably longer, with mean values of 90 and 55.2 ms for *Clockwise* and *Anticlockwise* perturbations, respectively, and a mean pairwise difference of 33.6 ms. Most of the participants’ CR latencies (*Clockwise*: 10/12; *Anticlockwise*: 12/12) were shorter than 110 ms, hence too fast to be under voluntary control. The onset latencies of CRs to *Clockwise* perturbations (90 ms) were longer than the expected for spinal SLRs (<60 ms) and fast transcallosal responses (70 ms), suggesting the mediation of supraspinal and transcortical pathways. However, the average onset latency of CRs to *Anticlockwise* perturbations (55.2 ms) was shorter, with most (10/12) participants having CRs with latencies <60 ms and all of them (12/12) having CRs with latencies <70 ms, suggesting the main involvement of subcortical pathways.

## DISCUSSION

We have shown that activation of sensory afferents in one hand, either through a mechanical perturbation to the index finger, or through nerve stimulation at the wrist, can elicit a CR in the contralateral intrinsic hand muscle. The earliest component seen with either type of afferent activation was a suppression, typically <70 ms. We also showed that CRs were sensitive to the direction of the perturbation in the contralateral finger, but they were relatively insensitive to the amplitude of the perturbation, as well as to the bimanual requirements of the task. Collectively, these results suggest that CRs in intrinsic hand muscles during bimanual coordination are mediated by both subcortical and transcortical pathways.

### Sensitivity of CRs in Hand Muscles to Task Demands

Similar to LLRs, CRs during more proximal upper limb movements can be modified in a task-dependent manner. This is readily exemplified in one/two cursor tasks where perturbations given during trials in which each hand controls the position of a separate cursor tend to cause weaker or no CRs compared with trials where both hands share control of a single cursor (Mutha and Sainburg 2009; Omrani et al. 2013). In a task by Dimitriou and collaborators (2012), participants were asked to control a virtual “tray,” and the response of the arms to either unimanual or bimanual perturbations was shown to depend on the nature of the control of the virtual tray: the same mechanical perturbation could produce very different responses (Dimitriou et al. 2012).

While we found that CRs in finger muscles were indeed sensitive to the direction of the perturbation in the contralateral finger, our results did not find that finger CRs are particularly sensitive to the bimanual requirements of the task: we observed that the amplitude of CRs in the unperturbed finger was comparable while controlling the cursor in the task-relevant (*Linked* control, *experiment 1A*) and task-irrelevant (*Orthogonal* control, *experiment 2*) dimension. We must consider whether our definition of task-relevant and -irrelevant dimensions was valid in relation to the way participants engaged with the task. A two-cursor approach might help clarify this, but given previous work on bimanual grip tasks, substantially different results are unlikely. During a bimanual grip of a real or virtual object, the grip force between the two hands is coupled: when the object undergoes a unilateral downward perturbation (an increase in load) on one hand, both hands show an increase in grip force (Bracewell et al. 2003). Most importantly, this coupling remains even when participants were gripping a separate object in each hand (White et al., 2008), suggesting a coupling that is less flexible than that observed for proximal muscles. In this respect, our results align with the observations of Bracewell et al. (2003) and White et al. (2008). The order of the experiments (*Linked* control, *experiment 1A*; *Orthogonal* control, *experiment 2*) was randomized between participants; therefore, our results cannot be explained by participants “locking” into the response pattern most appropriate for *Linked*, as opposed to *Orthogonal* control. Recent work has also shown that when participants were allowed a longer time interval to respond to a perturbation, they tend to “shift” the task dependency of LLR responses toward the belated voluntary epochs (Omrani et al. 2013). We did not test variable hold times; therefore, we cannot exclude shifts toward voluntary epochs being a confound factor in our task. However, even with the time interval allowed in our experiments, the score during perturbation trials was much lower than during trials with no perturbation, indicating that participants responded to perturbations promptly.

In many everyday actions, such as opening a bag of crisps or carrying a tray, both hands are coordinated to perform mirrored-like movements. In consequence, most previous work has examined CRs using analogous experimental paradigms (e.g., Diedrichsen and Dowling 2009; Ohki and Johansson 1999; White and Diedrichsen 2013), where the control signals do not differ between the two upper limbs. However, not all everyday actions are coordinated in a mirror-like fashion; think about unscrewing the lid off a bottle, for example. In our study, the control signals are different between both hands (left, force vs. right, position) to simulate these cases. Whether the task flexibility of distal CRs depends on the similarity of the control policy between both hands needs to be explicitly tested in further work, but altogether, our results show that the previously reported lack of flexibility in bimanual finger interactions can also be seen in intrinsic hand muscles.

### Distal Source of Sensory Feedback for CRs

Although our study aimed to localize the perturbation to the index finger, it is almost certain that muscles in the forearm with tendinous insertions onto the index finger were also perturbed, contributing to the observed CRs during the task. However, we have shown that activation of afferents distal to the wrist via electrical stimulation is sufficient to elicit CRs in the 1DI muscle that resemble CRs to mechanical perturbations, both displaying early suppression (*experiment 3*, Fig. 7).

Our stimulation setup used a distal cathode that was optimal for direct activation of motoneuron axons innervating hand muscles. A proximal anode allowed us to cause a weak twitch in intrinsic hand muscles with minimal activation of forearm muscles. Through anodal block, we could also reduce direct afferent activation, although we did not measure it in our setup. Therefore, we cannot exclude that direct afferent rather than reafferent volleys might explain the observed shorter onset latency of CRs to electrical stimulation compared with mechanical perturbations (see Fig. 7; 44 vs. 59 ms), but even if that was the case, the activated afferents were still distal to the wrist.

Interestingly, median nerve stimulation seemed much more effective at eliciting CRs than ulnar stimulation (Fig. 7). The cutaneous innervation via the median nerve includes the palmar surface of the hand and the fingertips of digits 1 to 3 (as well as part of digit 4), which are functionally the most relevant to movements involving the 1DI and AbPB. This might suggest that cutaneous afferents contributed to the observed CRs in intrinsic hand muscles. Cutaneous inputs are a well-established source of input to commissural interneurons in the lumbar cord (Edgley and Jankowska 1987; Harrison et al. 1986; Jankowska and Noga 1990), many of which are inhibitory (Bannatyne et al. 2003, 2009). In humans, cutaneous stimulation alone is sufficient to elicit CRs during grip (Ohki and Johansson 1999).

A cutaneous contribution might also explain the lack of CRs sensitivity to perturbation amplitude (*experiment 1B*). If CRs were mediated through pathways relying on low threshold cutaneous afferents, and these cutaneous afferents were activated near maximally with *Weak* perturbations, *Strong* perturbations could not produce substantial increase of CRs in already saturated pathways. This requires further examination, for example, by varying the intensity of mechanical perturbations and electrical stimulation over a greater range of values.

Interestingly, the earliest response of feline lumbar spinal cord interneurons to contralateral nerve stimulation plateaued at a stimulation intensity consistent with low threshold cutaneous and group Ia afferent activation (Harrison et al. 1986). Stimulating with a stronger intensity did produce greater activation at longer latencies, but the earliest response remained unchanged.

Regardless of the exact source of the sensory response, our results show that CRs can be elicited in an intrinsic hand muscle from activation of contralateral low threshold afferents, as previously shown for forearm muscles (Delwaide and Pepin 1991; Zehr et al. 2001).

### Neural Substrates for Crossed Responses in Hand Muscles

Although CRs to mechanical perturbations in the upper limb were reported more than 30 years ago (Marsden et al. 1981) and have been extensively studied ever since (Diedrichsen 2007; Diedrichsen and Dowling 2009; Diedrichsen and Gush 2009; Dimitriou et al. 2012; Mutha and Sainburg 2009; Ohki and Johansson 1999; Omrani et al. 2013), questions remain regarding their neural substrates. The noninvasive paradigms used in this study provide indirect evidence about plausible pathways.

Given the important role that M1 has in LLRs and the control of distal muscles, a likely candidate for mediating crossed responses is the corpus callosum, as it densely interconnects motor and premotor areas between the two cortical hemispheres. Transcallosal interactions are primarily inhibitory (Asanuma and Okuda 1962; Chowdhury et al. 1995; Chowdhury and Matsunami 2002; Hanajima et al. 2001; Reis et al. 2008), which aligns with our results. In our task, however, the observed CRs are unlikely to be transcallosal due to two factors. First, crossing the corpus callosum adds at least an ~10-ms conduction delay to CRs, as this is the earliest delay for observing transcallosal inhibitory effects in humans (Ferbert et al. 1992). For an intrinsic hand muscle such as 1DI, we expect an ipsilateral LLR to occur between 60 and 100 ms and for a transcallosal CR to occur at least from ~70 ms onwards. The onset latency for the inhibitory CRs to *Anticlockwise* perturbations was on average 55 ms in our data, with >75% of participants having an onset latency <60 ms.

Second, the hand representation in “caudal” M1 (Rathelot and Strick 2009) that directly innervates distal motoneurons has very sparse callosal connections (Rouiller et al. 1994). If finger CRs were mediated through transcallosal connections of premotor areas and/or rostral M1 (Gould et al. 1986; Jacobson and Trojanowski 1974; Karol and Pandya 1971; Liu et al. 2002; Marconi et al. 2003; Pandya et al. 1971), we would expect them to occur at even longer latencies. Therefore, a transcallosal pathway seems a plausible route for the late facilitatory CRs to *Clockwise* perturbations observed in our study but not for the short inhibitory CRs.

This suggests that subcortical pathways contribute to the CRs seen in 1DI. There are multiple motor structures in the brainstem that receive sensory inputs from the periphery, including the cerebellum (Allen et al. 1977; Oscarsson 1965), superior colliculus (Massopust et al. 1985), and reticular formation (RF) (Soteropoulos et al. 2012), which could be involved. Of these, the RF has the strongest and bilateral connectivity with limb circuits in the spinal cord (Davidson and Buford 2006; Davidson et al. 2007; Peterson 1979; Peterson et al. 1979), directly connecting with motoneurons innervating intrinsic hand muscles (Riddle et al. 2009). The RF is involved in a variety of proximal motor functions including posture and locomotion, but recent work in primates has highlighted its involvement in goal directed movements of the arm (Buford and Davidson 2004) and hand (Soteropoulos et al. 2012). The RF can also access inhibitory spinal motor systems (Rudomín et al. 1983; Takakusaki et al. 2001) and also has direct inhibitory projections to the spinal cord (Du Beau et al. 2012): this makes it a plausible candidate for mediating the inhibitory CRs reported here.

A final possibility to be considered is the spinal cord. It has been known for some time that sensory afferents can affect contralateral spinal circuits (Holmqvist 1961a, 1961b; Perl 1958), and since then, extensive work done in the lumbar cord of either cat or rodent models (Butt and Kiehn 2003; Gauthier and Rossignol 1981; Jankowska 2008; Kiehn and Butt 2003; Quinlan and Kiehn 2007) has expanded our understanding on the bilateral organization of the spinal cord (Maxwell and Soteropoulos 2019). Although noxious stimulation has been known to activate spinal circuits bilaterally, for example, the crossed extensor reflex (Sherrington 1910), nonnoxious afferents from cutaneous group I and group II receptors also form a very potent source of inputs to commissural cells (Edgley et al. 2003; Harrison et al. 1986). The spinal commissural system has a very diverse neuroanatomical organization and consists of both excitatory and inhibitory cells, contacts contralateral interneurons and motorneurons, and has both a segmental and propriospinal connectivity (Molenaar and Kuypers 1978).

Evidence for commissural interactions in humans are well established for the lower limb (Côté et al. 2018), either to mechanical perturbation (Stevenson et al. 2015) or nerve stimulation (Gervasio et al. 2013; Hanna-Boutros et al. 2014; Stubbs and Mrachacz-Kersting 2009; Stubbs et al. 2011a, 2011b): in these studies the primary interaction between the limbs is an inhibition and the spinal CRs were also sensitive to the direction of the perturbation in the contralateral knee, as well as scaling to the background level of muscle activity (Stevenson et al. 2015). There is also evidence for commissural interactions in the upper limb, whereby similarly to the lower limb, inhibition is observed (Delwaide and Pepin 1991; Delwaide et al. 1988; Sabatino et al. 1992). More recently, trains of stimuli have been used to elicit interlimb CRs in forearm muscles (Carroll et al. 2005; Thomas et al. 2018; Zehr et al. 2001), although the pathway mediating these responses is not yet universally agreed upon. Some of our recent animal work has highlighted that commissural interactions can also be seen in intrinsic hand muscles as well (Soteropoulos et al. 2013). The latency of CRs, measured as changes in grip force, is also suggestive of spinal component (Ohki and Johansson 1999; White et al. 2008).

If the CRs reported here are a result of spinal pathways, then their latency should be comparable to that of the short latency responses seen in the R1DI (~33 ms), with an additional transspinal delay. The expected latency for spinally mediated EMG responses is based on the conduction delay across the monosynaptic stretch reflex, which is the fastest and most direct route. However, additional (and slower) sensory receptors, such as cutaneous and group II afferents, are also likely to be activated, all of which can have potent effects on motoneurons through indirect and oligo-synaptic pathways (Baudry et al. 2009; Chaix et al. 1997; Egger and Wall 1971; Marchand-Pauvert et al. 2000; Marque et al. 2001; Simonetta-Moreau et al. 1999), with added delays. For the lumbar cord in humans, the reported transspinal delay is in the range of 13–20 ms for mechanical perturbations (Stevenson et al. 2015). If we assume a comparable delay for the similarly sized cervical cord, CR latencies <63 ms would be compatible with a path through cervical segmental and propriospinal commissural circuits: 85% of the earliest CR latencies in the participants reported in our study were shorter than that. For the earliest CRs, a spinal pathway is thus a plausible candidate.

### Functional Considerations and Conclusions

The earliest components of the finger muscle CRs reported here are likely to be mediated subcortically, most likely at the level of the spinal cord. Although such pathways would be typically deemed as “low level” and fully automated, they could nonetheless play an important role during voluntary movements, many of which are bilateral during daily movements. This is exemplified by the propriospinal system, also located within the spinal cord. Although under tight descending control (Alstermark et al. 1984), this system still plays an important role in reaching-to-grasp movements both in health (Giboin et al. 2012; Kinoshita et al. 2012; Nicolas et al. 2001; Pierrot-Deseilligny and Marchand-Pauvert 2002) and following motor damage (Alstermark et al. 2011; Isa et al. 2006; Nishimura and Isa 2011; Tohyama et al. 2017). Similarly, the brainstem RF, often thought of as a simple postural control system, has been recently shown to play a role during voluntary movements with the upper limb (Buford and Davidson 2004; Schepens et al. 2008; Soteropoulos et al. 2012), particularly after corticospinal damage (Ellis et al. 2012; Schwerin et al. 2008; Zaaimi et al. 2012).

The functional role of the CRs in intrinsic hand muscles is as yet unclear, but at least for this task, they do not seem to be operating under the more flexible control policies commonly reported for more proximal muscles and movements (Crevecoeur et al. 2016; Omrani et al. 2013; Pruszynski et al. 2014; Pruszynski and Scott 2012). The CRs seemed to mirror the responses seen in the perturbed muscle. This low-level mirroring may facilitate a rapid increased grip force in response to an object slipping from grasp (Ohki and Johansson 1999; White et al. 2008) in the case of the slower facilitatory CRs. However, the earlier inhibitory response is difficult to explain within this context. Alternatively, it could be an echo from activation of brainstem or spinal commissural circuits involved in mediating alternate activation of muscles across the midline, often seen during locomotion and climbing: sensory feedback from grasping from one hand, if gated appropriately, could instead facilitate termination of grasping in the contralateral hand and help maintain the alternating pattern of activity in homologous muscles between limbs. Further studies examining CRs in intrinsic hand muscles during more varied bimanual and unimanual tasks, especially including whole arm movements, might help clarify the functional role of these responses and the potential contribution of subcortical structures in CR. Studies on the neural structures mediating distal CRs could also benefit from animal work, as these subcortical structures are much harder to access noninvasively in humans.

Spinally mediated CRs may also play a role following motor damage such as stroke and spinal cord injury (SCI). Beyond the initial injury that causes the loss of descending drive to the spinal cord, there is evidence of long-term changes in many spinal circuits, including those mediating CRs following stroke (Stubbs et al. 2012; Zehr and Loadman 2012) and SCI (Calancie et al. 1996, 2002, 2005). Such changes could be responsible for some of the difficulties affecting patients during bimanual movements, where using both hands together can have a detrimental impact on the performance of the less affected upper limb (stroke: Rose and Winstein 2005, Rice and Newell 2004, SCI: Calabro and Perez 2016). Whether the CRs reported here play a role in shaping, either positively or negatively, the recovery of hand function following motor damage remains to be determined.

## GRANTS

This work was supported by a New Investigator Award from the Medical Research Council (UK) (to D. S. Soteropoulos).

## DISCLOSURES

No conflicts of interest, financial or otherwise, are declared by the authors.

## AUTHOR CONTRIBUTIONS

K.Y.W.K. performed experiments; K.Y.W.K. and D.S.S. analyzed data; K.Y.W.K., F.G., and D.S.S. interpreted results of experiments; K.Y.W.K. and F.G. approved final version of manuscript; D.S.S. conceived and designed research; D.S.S. prepared figures; D.S.S. drafted manuscript; F.G. and D.S.S. edited and revised manuscript.
